# Identifying physiological tissue niches that support the HIV reservoir in T cells

**DOI:** 10.1128/mbio.02053-23

**Published:** 2023-09-25

**Authors:** Nnamdi Ikeogu, Oluwaseun Ajibola, Romaniya Zayats, Thomas T. Murooka

**Affiliations:** 1 Department of Immunology, Rady Faculty of Health Sciences, University of Manitoba, Winnipeg, Manitoba, Canada; 2 Department of Medical Microbiology and Infectious Diseases, Rady Faculty of Health Sciences, University of Manitoba, Winnipeg, Manitoba, Canada; Albert Einstein College of Medicine, Bronx, New York, USA

**Keywords:** HIV-1, lymph node, cell migration, HIV latency, cell-cell interaction

## Abstract

Successful antiretroviral therapy (ART) can efficiently suppress Human Immunodeficiency Virus-1 (HIV-1) replication to undetectable levels, but rare populations of infected memory CD4^+^ T cells continue to persist, complicating viral eradication efforts. Memory T cells utilize distinct homing and adhesion molecules to enter, exit, or establish residence at diverse tissue sites, integrating cellular and environmental cues that maintain homeostasis and life-long protection against pathogens. Critical roles for T cell receptor and cytokine signals driving clonal expansion and memory generation during immunity generation are well established, but whether HIV-infected T cells can utilize similar mechanisms for their own long-term survival is unclear. How infected, but transcriptionally silent T cells maintain their recirculation potential through blood and peripheral tissues, or whether they acquire new capabilities to establish unique peripheral tissue niches, is also not well understood. In this review, we will discuss the cellular and molecular cues that are important for memory T cell homeostasis and highlight opportunities for HIV to hijack normal immunological processes to establish long-term viral persistence.

## INTRODUCTION

Human Immunodeficiency Virus-1 (HIV-1, herein referred to as HIV) preferentially infects and depletes CD4^+^ T cells, thereby reducing host adaptive immunity if left unchecked (1). While current antiretroviral therapies (ARTs) can suppress viral loads to undetectable levels, ART interruption leads to a predictable rebound in viremia to pre-treatment levels in most individuals (2). Rebounding virus originates from a pool of infected cells that are established early (3) and continues to persist in ART-treated people living with HIV (PLWH) (4). The inability of current treatment regimens to completely eliminate residual infected cells remains the most significant barrier to achieving HIV cure and continues to be an area of intense investigation (5). From the virus point of view, infecting and establishing latency within memory CD4^+^ T cells offer a number of advantages. First, infected T cells remain motile (6 – 8), providing a means for virus to disseminate widely while avoiding recognition by extracellular immune factors. Infected T cells that enter secondary lymphoid organs can physically interact with other susceptible cells, forming cell-cell contacts that greatly enhance viral spread between cells (6, 8). Another advantage hinges on the fact that memory CD4^+^ T cells are long-lived, acquiring survival and proliferative signals through cell-cell interactions and homeostatic cytokines during their physiological transit through lymphoid and non-lymphoid tissues (9). Thus, suppressing viral replication to restore normal T cell function may allow cells harboring replication-competent provirus to access the same survival and proliferative cues, ensuring long-term longevity of the viral reservoir (9). Here, we will review lymphoid tissue niches that are instrumental for the long-term survival and function of memory CD4^+^ T cells, and discuss how HIV may co-opt physiological cues to facilitate the establishment and maintenance of the HIV reservoir. Dismantling key cellular and molecular determinants that contribute to the longevity of the viral reservoir may provide novel insights toward reducing reservoir size under ART suppression.

### The presence of HIV reservoirs are a major barrier to cure

The development and improved accessibility of ART have significantly decreased HIV incidence and mortality rates worldwide (10), with ART access increasing by over sevenfold between 2006 and 2017, leading to a ~50% reduction in global mortality over this period (10). A standard antiretroviral therapy regimen consists of two or more drugs, collectively referred to as highly active ART and these function to block HIV entry, replication, and/or maturation, resulting in undetectable viremia and partially restoring CD4^+^ T cell count in PLWH (11, 12). While the discovery and deployment of ART have been an astounding success in reducing HIV transmission and prolonging the lifespan of PLWH, drug regimens need to be closely monitored to ensure long-term therapy efficacy and adherence (10, 13). Reported successes with allogeneic bone marrow transplantations that achieved durable, undetectable viremia in the absence of ART are encouraging (14 – 18), but this procedure is associated with significant cost and/or risk. Therefore, allogeneic bone marrow transplantation does not represent a widely available HIV cure strategy.

While current antiretroviral drug combinations are effective in reducing viremia to undetectable levels, viral replication rebounds to pre-treatment levels upon therapy interruption in most individuals. This has significant clinical implications, leading to treatment failure, increased susceptibility to illnesses, and higher risk of transmission to others (19). Antiretroviral drugs also do not eliminate a population of persistently infected cells harboring replication competent but transcriptionally silent provirus, a possible source of viral rebound upon ART interruption (20 – 23). The half-life of the HIV reservoir is reported to be 43.9 months or ~3.7 years (24, 25), indicating that its natural decay would take over 73 years, thus not achievable in one’s lifetime (23). Importantly, initiation of antiviral therapy during the earliest stage of acute HIV infection (Fiebig I) is not sufficient to prevent viral rebound following analytical treatment interruption, indicating that the HIV reservoir is established shortly after infection (26). Similar findings were reported in SIV-infected rhesus macaques, where ART treatment at day 3 post-challenge reduced plasma viremia and detection of proviral DNA in secondary lymphoid tissues but led to viral rebound in all animals upon ART discontinuation following a 24-week treatment regimen (3). Follow-up studies subsequently showed that the viral reservoir was established within 1–2 days following exposure (27). Studies using humanized BLT (***b*** one marrow, ***l*** iver, and ***t*** hymus) mice also display rapid viral rebound kinetics after 2 weeks of ART interruption (28, 29). ART-suppressed BLT mice continue to harbor latently infected resting human CD4^+^ T cells that can be induced to produce HIV *ex vivo* (28, 29). These studies collectively show that long-lived HIV reservoirs are established soon after transmission, presenting a considerable challenge for therapeutic interventions to prevent the establishment of the viral reservoir.

Establishing latency is a commonly used viral strategy to evade clearance by the host immune response and maintain infection chronicity (30). In the case of HIV, targeting CD4^+^ T cells not only disrupts anti-HIV adaptive immunity, but allows access to highly migratory cells that can enter and exit almost all tissues in the body (6, 7). We and others have previously shown that HIV-infected T cells retain their migratory phenotype, albeit at reduced speeds, and drive systemic viral dissemination to distant tissues during the early acute phase of infection (6, 7). This also allows HIV-infected T cells to access other target cells within lymphoid organs through cell-cell interactions (8, 31). Another favorable feature of T cells is their ability to transition between activated and quiescent states that directly modulate viral replication potential (32 – 34). HIV latency in T cells can be broadly classified into two mechanisms: pre- and post-integration latency (35). During pre-integration latency, unintegrated viral RNA remains in the host cytoplasm for days, where the viral genome forms the pre-integration complex (35 – 38). The low metabolic state of resting CD4^+^ T cells prevents viral integration into the host genome to maintain a state of pre-integration latency (39, 40). In contrast, post-integration latency may be established when productively infected CD4^+^ T cells revert to a quiescent state, thereby reducing the availability of host factors required for efficient viral transcription and replication, such as NFAT and NFκB (39 – 42). Other described mechanisms of HIV latency in T cells include the absence of Tat expression and other associated host factors that promote transcriptional elongation (43) and transcription interference (44). Strategies to reinvigorate viral transcription as part of a “shock and kill” (45 – 48) approach to purge the viral reservoir have been met with limited clinical success (21, 49), with recent studies demonstrating that proviral integration site and associated epigenetic regulation greatly impact the susceptibility of proviruses to LRAs (50). Recent efforts are focused on combining different approaches to improve the elimination of infected cells, such as the use of bcl-2 antagonists to improve CTL killing of infected T cells (51).

### Secondary lymphoid organs coordinate adaptive immunity generation

The thymus and bone marrow provide specialized niches for lymphocyte development, whereas secondary lymphoid organs coordinate the generation of protective adaptive immunity (52). Lymph nodes (LNs) are strategically dispersed throughout the body and are continuously surveyed by leukocytes circulating through blood, lymph, and peripheral tissues to maximize the generation of rare, pathogen-specific responses in a timely manner (53 – 55). An important structural characteristic of lymph nodes is the presence of specialized vascular and lymphatic systems that support the sampling of tissue-draining lymph by T and B lymphocytes (53 – 56). The afferent lymph-draining tissues enter regional lymph nodes through the subcapsular sinus (SCS) that are lined with CD169^+^ SCS macrophages (53 – 56). These macrophages function as “fly traps” through their continual sampling of incoming lymph and presenting/transferring antigens, while also serving as targets of infections (53 – 56). The LN cortex, just beneath the subcapsular space, is divided into an outer (follicular cortex) and an inner cortex (paracortex) (53 – 56). The follicular cortex (or B cell zone) is rich in follicular dendritic cells (FDCs), CD4^+^ follicular T helper cells, and B cells (57). Naïve and memory T cells enter lymph nodes via the high endothelial venules (HEVs) and are guided into the paracortex (or the T cell zone) by chemokines CCL21 and CCL19 as they rapidly migrate along fibroblastic reticular cell (FRC) networks in search of cognate antigen (57). Peripheral tissue-derived dendritic cells (DCs) transmigrate through the SCS floor via the afferent lymphatics, and are also guided into the paracortical region by lymph node homing chemokines produced by FRCs (58). Migrating T cells engage with a stable network of DCs along FRCs that, upon cognate antigen recognition, results in T cell activation, proliferation, and differentiation over the next few days (59, 60). In the absence of antigenic stimulation, T cells actively leave the lymph node via cortical sinuses in an S_1_PR1-mediated, multistep process, and enter other lymph nodes (61, 62).

#### Role of tonic TCR signaling in long-term memory T cell survival

The contribution of tonic TCR signals toward the maintenance of memory CD4^+^ T cells is not clearly defined. Takeda et al. showed that following engraftment of fetal thymi from donor wildtype mice into thymectomized Rag2^−/−^MHC-II^+/+^ or Rag2^−/−^MHC-II^−/−^ recipients, newly generated (donor) CD4^+^ T cells proliferated similarly amongst the two recipient groups. However, over the course of 6 months, there was a decline in the number of donor CD4^+^ T cells in Rag2^−/−^MHC-II^−/−^ recipients. These observations established the important contribution of MHC-II-mediated tonic TCR signaling to the survival of memory CD4^+^ T cells *in vivo* (63). Hataye et al. also showed that TCR:MHC-II interactions were crucial for naïve and memory CD4^+^ T cell survival *in vivo* by demonstrating that competition for MHC-derived signals regulated clonal expansion of transferred T cells (64). In contrast, Swain et al. reported high survival rates of adoptively transferred, exogenously activated murine CD4^+^ TCR transgenic T cells into MHC-II deficient recipients (65), although these studies were performed using lymphopenic mice lacking endogenous T cells. The limitation of this approach is that transferred T cells can undergo homeostatic proliferation independent of MHC-II-derived signals, but even under these conditions, expanded CD4^+^ T cells lacked normal expression levels of some cell surface markers, such as CD44 as they aged, indicating dysregulation of cellular functions (63). Indeed, tonic TCR signaling was dispensable for survival in another study, but strong functional defects upon antigen re-encounter were observed (66). DC:T cell interactions in the absence of cognate antigen induced a basal level of T cell activation which was required for optimal responses against subsequent antigenic stimulation *in vivo* (67). Thus, while the contribution of continual TCR:MHC-II contacts to the long-term survival of memory CD4^+^ T cells *in vivo* is debatable, tonic signaling at steady-state is crucial for maintaining high functional quality of memory T cells upon secondary challenge.

#### Contribution of antigenic stimulation in memory T cell maintenance

CD4^+^ T cell activation is followed by differentiation and expansion into effector T cell subsets, after which memory T cell pools are generated (68). Compared to primary responding cells, memory T cells undergo rapid re-activation and generally require lower doses of antigen and co-stimulation (69, 70). Several studies have demonstrated that, repeated antigen encounter help promote memory CD4^+^ T cell survival, thereby contributing to the maintenance of the overall memory T cell pool (71, 72). Gray et al. showed that in order for memory CD4^+^ T cells to maintain their capacity to provide B cell help, repeated antigen exposure was a critical requirement (73). Recent SARS-CoV-2 infection studies also indicate the importance of repeated antigenic boosting to maintain functional memory CD4^+^ T cell responses. Using the HLA-DRB1*15:01/_S751_ tetramer to track spike-specific CD4^+^ T cells, Wragg et al. showed that primary infection, or vaccination, induced robust S_751_-specific CXCR5^−^ and circulating T follicular helper (cT_FH_) cell memory responses. Furthermore, secondary viral exposure induced recall of CD4^+^ T cells with a transitory CXCR3^+^ phenotype and drove expansion of cT_FH_ cells that transiently expressed ICOS, CD38, and PD-1 (74). Similarly, Dan et al. showed that SARS-CoV-2 specific CD4^+^ T cell memory was detectable in 93% of individuals sampled 1 month after infection, which was maintained at high levels (92%) after 6–8 months post-infection, including SARS-CoV-2 Spike-specific memory T follicular helper (T_FH_) cells (75). These findings support the notion that repeated antigenic stimulation helps maintain the number and functionality of virus-specific memory CD4^+^ T cell pools against re-infections.

#### Homeostatic cytokines and memory T cell responses

In conjunction with antigen re-encounter, homeostatic cytokines IL-7 and IL-15 also play a supportive role in maintaining memory T cell survival (76 – 78). Naïve and memory T cells retain high expression of IL-7R (79), where IL-7 engagement promotes cell survival by upregulating several anti-apoptotic signaling pathways (80). The size of the T cell compartment in lymphoid organs is also regulated by FRC-derived IL-7 production (81) and acts as a critical long-term survival signal for T cells (82). In non-lymphoid tissues, IL-7 production by epithelial cells can also help maintain tissue-resident memory T cell pools (83). IL-7Rα expression is lost upon T cell activation through Foxo1, which itself is negatively regulated downstream of the TCR (84), but is re-expressed as antigen load decreases over time (85). Importantly, IL-7R^hi^ effector cells preferentially give rise to long-lived memory cells that confer protective immunity, indicating that IL-7 is involved in the transition from effector to memory T cells as antigen load wanes (85). Recombinant simian IL-7 (rsIL-7) therapy given to aged rhesus macaques resulted in doubling of central memory T cell numbers in the periphery, whereas no beneficial effects were observed within the naïve T cell compartment (78). IL-7 therapy also led to a rapid expansion of the memory CD4^+^ T cell compartment in ART-suppressed PLWH, resulting in an increase in the size of the HIV T cell reservoir (86). Conversely, therapeutic blockade of IL-7R reduced the frequency of recurrent, CD4^+^ T cell-driven skin inflammation in rhesus macaques, which was associated with their reduced frequency and diminished IFNγ production after antigenic re-stimulation *ex vivo* (87). These studies collectively illustrate that therapeutic IL-7 administration can manipulate memory CD4^+^ T cell frequency and function *in vivo*, with either beneficial or detrimental outcomes depending on the disease context.

### Cellular and tissue reservoirs of HIV during ART suppression

HIV can productively infect cells of myeloid lineages, including macrophages, monocytes, and immature dendritic cells (88 – 90), but infected memory CD4^+^ T cell subsets are regarded as the major component of the long-lived viral reservoir (91 – 93). While naïve T cells can contain integrated HIV DNA and may contribute to viral pathogenesis (94), memory T cell subsets harbor the majority of the total proviral burden under ART suppression (92). Initial characterization of the T cell reservoir showed that integrated provirus was enriched in central memory (T_CM_) and transitional memory (T_TM_) CD4^+^ T cells in virally suppressed PLWH (92). Recent advances in genetic research technologies have allowed researchers to perform high-resolution sequencing analysis of integrated, replication-competent HIV, where effector memory T cell (T_EM_) subsets have been shown to contain the highest proportion of inducible provirus, suggesting that cell proliferation is an important driver of T cell reservoir maintenance (93, 95). Interestingly, skewed distribution of the HIV reservoir from long-lived T_CM_ toward shorter-lived T_EM_ and T_TM_ are associated with viral control in elite controllers (96, 97). It is important to note that T_EM_ harboring latent HIV can persist for decades under ART, underscoring the notion that significant inter-individual differences in the extent to which proviruses persist within T cell subsets exist (98). Given the wide range of lymphoid and non-lymphoid tissues accessed by both circulating T_CM_ and T_EM_ cells, it is perhaps not surprising that there are many anatomical sites that contribute to viral rebound after ART interruption (95).

Distinct from migratory T_CM_ and T_EM_ subsets, tissue-resident memory T cells (T_RM_) display a unique transcriptional profile, reside at various tissue sites, and are non-circulating. Human T_RM_ in lymphoid and mucosal tissues are largely confined to the CD69^+^ T cell population, with CD103 co-expression further defining CD8 T_RM_, similar to those described in mice (99 – 101). CD69 expression facilitates degradation of S_1_PR1 (which faciliates T cell egress from secondary lymphoid organs) and along with reduced CCR7 and CD62L expression help define the non-circulating, tissue-resident phenotype of T_RM_ cells (99, 102). T_RM_ are ideally placed to respond to site-specific infections through the rapid production of inflammatory cytokines that subsequently help recruit and initiate local innate and adaptive immune responses (99, 103 – 106). Comprehensive phenotypic analysis shows that human T_RM_ cells are maintained throughout life in many organs, particularly at mucosal sites (100, 107) including cervical mucosal T_RM_ cells that also express high levels of CCR5 (108). Indeed, the latter study reported that HIV preferentially infected CD4^+^ T_RM_ T cells compared to non-T_RM_ cells in cervical explants and contained the highest proportion of viral DNA among T cell populations (108). Interestingly, Reuschl et al. showed that HIV transmission through cell-cell interactions promoted a T_RM_ phenotype in infected cells that was enhanced by IL-7. The HIV accessory protein Vpr was shown to drive a transcriptional reprogramming of resting T cells towards a T_RM_ phenotype, defined by upregulation of CD69, CD49a, PD-1, CD101, CXCR6, and Blimp-1 (109). These studies collectively argue that T_RM_ subsets may represent an important population targeted by HIV, either through direct infection or through induction of a T_RM_-like phenotype. Some overlap in transcriptional profiles that define T_RM_ and those that harbor latent provirus (e.g., PD-1) support the possibility that infected T_RM_ cells contribute to HIV persistence in tissues (99, 110 – 112).

**Fig 1:**
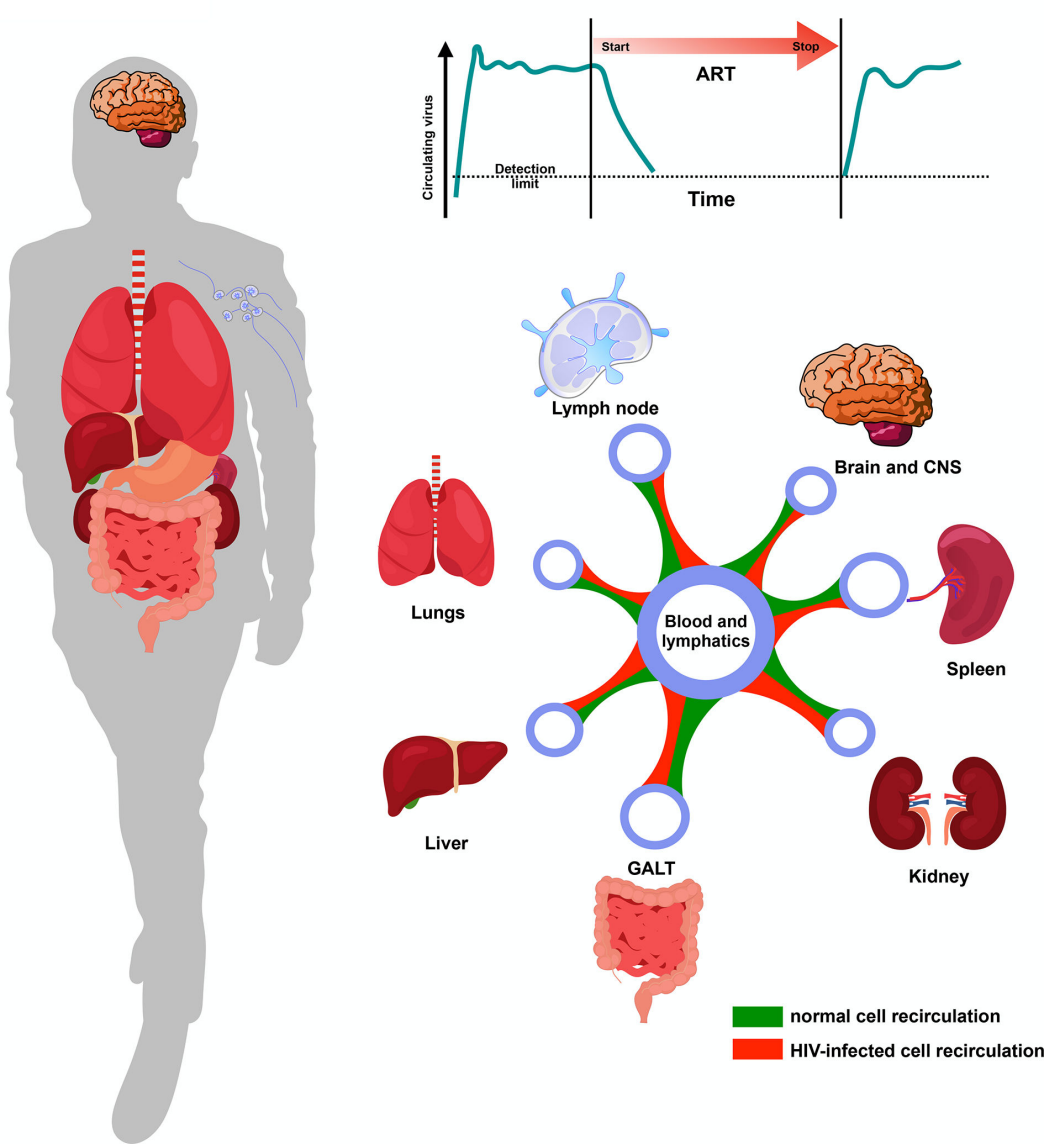
The interconnectivity of the latent HIV reservoir across different anatomical sites. The latent HIV reservoir in tissues is established soon after viral transmission and is not eliminated with current ART regimens. The clonal expansion of HIV-infected T cells and their broad distribution across various anatomical sites during ART suppression is depicted, with the circulatory and lymphatic systems acting as conduits for their recirculation through different organs. Green and red lines between the different compartments represent parallels in recirculatory networks between normal and HIV-infected cells, respectively. HIV rebound upon ART interruption can originate from diverse cellular and tissue reservoirs.

Genetically identical viral sequences were found within the memory T cell compartment, but no one dominant tissue source for rebounding virus was observed (95). Near full-length Env sequencing from individuals that participated in the Last Gift program also showed that viral DNA was present in many organs sampled, with little evidence for genetic compartmentalization (113, 114) (Fig. 1). These observations are in line with evidence for active viral exchange between tissues, likely through peripheral blood, resulting in similar distribution of reservoir virus during suppressive ART (95, 115). HIV^+^ individuals who initiated cART early after infection generally had lower levels of cell-associated HIV DNA in their tissues compared to those in PBMC, with highest levels in lymphoid tissues including the GALT and lymph node (115, 116). Similarly, the blood, gut, and lymphoid tissues are key players in viral dissemination. While the gut and lymphoid tissues serve as hubs for viral dissemination, the circulatory system is the likely conduit for this systemic spread (117). Post-mortem tissue analysis from ART-treated individuals revealed the presence of HIV DNA levels in many tissue samples, including the lymph node, lung, liver, aorta, spleen, and kidney (118). Notably, these and other studies (113) identified the brain as an important reservoir tissue, with signs of pathology observed in all individuals (118). A recent study observed latently infected T cells in the CNS as a source of viral rebound and clonal reservoir expansion during therapy interruption (119)

Studies in Simian Immunodeficiency Virus (SIV)-infected macaques, permits the sampling of a wide range of organs at specific time points during disease progression, to assess tissue sites that harbor SIV DNA and RNA under ART suppression (120). Prior to ART treatment, lymphoid organs contained >98% of SIV RNA^+^ and DNA^+^ cells, indicating vigorous, uncontrolled viral replication at these tissue sites (121). Upon ART treatment, substantial decrease in SIV RNA^+^ cells was observed, and all were localized within lymphoid tissues. Similar observations were made in early ART-treated rhesus macaques; at 1-week post-infection, viral DNA was detected in multiple tissues, whereas viral RNA expression was restricted anatomically to lymph nodes (121). Of all sampled tissues, the mesenteric lymph node contained the highest levels of cell-associated, replication-competent SIV DNA and contributed to viral rebound upon ART withdrawal (121, 122). Lymphoid organs also harbored infected cells capable of reactivating viremia in infected humanized BLT mice, leading to a 50-fold increase in the frequency of HIV RNA^+^p24^+^ cells (123). Under long-term ART, the gut may serve as another anatomical niche for viral reservoirs, as HIV DNA were found to be five to six times higher in the ileum compared to peripheral blood in individuals on ARTs for 10 years (124). Previous longitudinal assessment of HIV replication and viral evolution, between GALT and peripheral blood mononuclear cell (PBMC) compartments showed that the GALT was not the major contributor to viral rebound after ART interruption (125). However, other studies have demonstrated the importance of the gut reservoir to viral rebound following ART withdrawal (126).

### HIV co-opt signals in secondary lymphoid organs to establish long-term persistence

**Fig 2:**
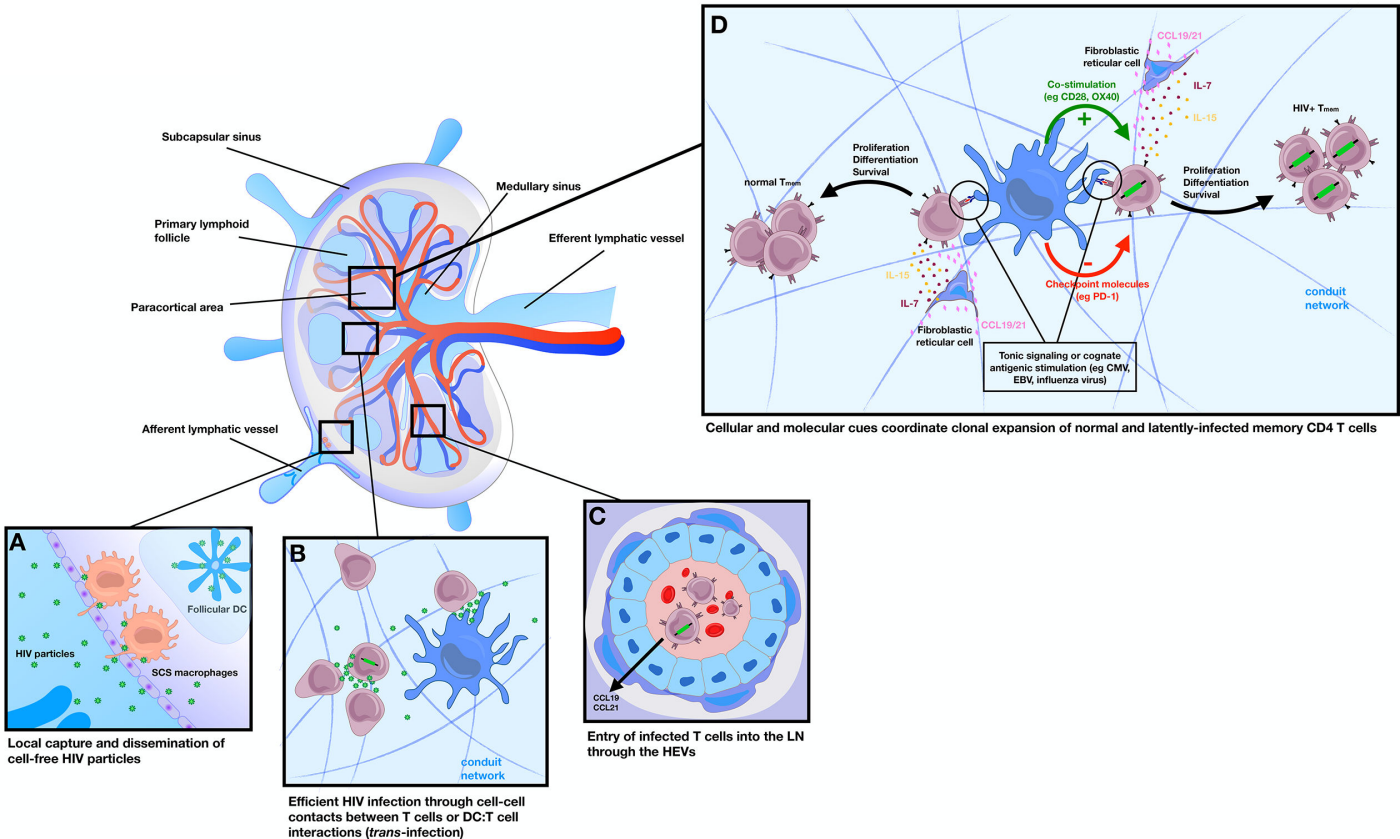
The lymph node as a major hub for HIV dissemination, latency generation, and clonal expansion of the HIV T cell reservoir. Lymph nodes are one of the most important tissue HIV reservoir sites, where their structural organization supports viral infection, pathogenesis, and latency. (**A**) CD169^+^ subcapsular sinus (SCS) macrophages capture cell-free HIV particles for local dissemination to T cells, whereas follicular dendritic cells (FDCs) retain infectious HIV particles within a non-degradative cycling compartment and disseminate virus to target CD4^+^ T cells. (**B**) In the paracortical region, efficient HIV spread occurs between T cells (virological synapse) or between HIV-captured DCs and T cells (*trans*-infection). (**C**) HIV-infected memory T cells enter the lymph node parenchyma via the high endothelial venules (HEVs) and are guided to the paracortical region by CCL19/21 gradients. (**D**) Access to cognate antigen, tonic TCR stimulation by resident dendritic cells (DCs) and other proliferative/survival signals (e.g., IL-7 and IL-15) drive the clonal expansion of HIV-infected T cells using mechanisms analogous to normal memory T cell generation. Fine tuning of TCR signaling strength by co-stimulation and checkpoint molecule expression coordinates downstream survival and proliferative responses of HIV-infected T cells.

As described above, the tissue architecture and stromal/immune cell components of the lymph node help support the survival of naïve and central memory T cells through cytokine expression and physiological cell-cell interactions (86, 91). Given that central memory CD4^+^ T cells represent an important subset that harbor latent provirus with the capacity to re-establish infections (91 – 93, 127), their ability to recirculate through secondary lymphoid tissues may be instrumental to their long-term survival under ART (Fig. 2). FRC-derived IL-7 is abundant in the lymph node paracortex and known to enhance HIV infection (128) and support homeostatic proliferation of normal as well as latently infected T cells (86, 91, 129 – 131). The fact that IL-7 therapy led to an expansion of the HIV T cell reservoir illustrates their role in regulating reservoir size, likely within secondary lymphoid organs (86). Chemokines such as CCL19 and CCL21, which help position T cells within the lymph node T cell zone, promote latent infection in proliferating and non-proliferating T cells by enhancing viral entry and integration through modulation of the actin cortical cytoskeleton (132). The presence of immunomodulatory cytokines can induce a quiescent state in memory T cells harboring provirus by reducing access to cellular transcription factors needed for viral gene expression (86). Cytokines, such as TGFβ (133, 134) and IL-10 (135), have been shown to favor HIV latency by dampening cellular activation and HIV/SIV transcription in CD4^+^ T cell. These data suggest that the paracortical region of the lymph node may provide a favorable environment for latency generation and maintenance during physiological T cell transit, likely through a convergence of multiple inputs.

It is well documented that HIV can spread through both cell-free and cell-to-cell contacts (136), the latter predicted to be the dominant mode of transmission within tightly packed lymph nodes (6, 137). In one scenario, dendritic cells can capture viral particles through Siglec-1 and transfer virus at the site of DC:T cell contact, leading to efficient T cell infection (138, 139). Other prolonged T:T cells and macrophage:T cell contacts have been described, leading to increased HIV transmission rates at the site of cell-cell contact, and in some cases initiation of unique signaling cascades that promote virus replication in target cells (140 – 145). B cell follicles have long been considered an important source of new infections, where FDCs have the capacity to bind and retain intact HIV virions within a non-degradative cycling compartment for prolonged periods of time and transmit infectious particles to select CD4 T cells migrating along FDC networks (146 – 149). If such cell-cell contacts occur frequently and are conduits for rapid HIV transmission to susceptible T cells in lymphoid organs, it is conceivable that similar contacts are involved in mechanisms that regulate the generation and maintenance of HIV latency. Indeed, Agosto et al. showed that resting T cells infected through cell-cell contacts contained provirus that was harder to induce when compared to infection by cell-free HIV alone, suggesting that the context in which latency is established may dictate proviral inducibility and persistence (150). Cell-cell contacts also induce a TRM-like phenotype in infected cells that may promote HIV persistence in tissues (109), underscoring the potential for HIV to co-opt physiological processes to ensure long-term HIV reservoir establishment and maintenance.

Antigen specificity of CD4^+^ T cells also modulates their susceptibility to HIV infection (151). CD4^+^ T cells specific to HIV (152), *Mycobacterium tuberculosis* (153) as well as tetanus toxoid and *Candida albicans* (154) are preferentially targeted by HIV during acute infection, whereas cytomegalovirus (CMV)-specific CD4^+^ T cells are relatively protected through the autocrine production of CCR5 ligands (155). Emerging data from several groups also describe the role of antigen in the clonal expansion of latently infected T cells, producing daughter cells containing identical proviral sequences (129, 156 – 160). Mendoza et al. showed that CD4^+^ T cells containing defective or intact provirus were found in T cells responsive to HIV Gag, CMV, Epstein-Barr virus, and influenza virus-derived antigens (161). More recently, Simonetti et al. isolated CMV- and Gag-responding CD4^+^ T cells from ART-treated individuals and observed large clones harboring replication-competent provirus that was not explained by homeostatic proliferation or specific integration site effects (162). These studies indicated that repeated exposure to viral antigens may contribute to the longevity of the HIV reservoir through T cell proliferation, likely to occur within secondary lymphoid organs where the frequency of APC:T cell interactions are high, rather than ongoing viral replication under ART (156). HIV DNA^+^ memory CD4 T cells also display transcriptional signatures associated with the inhibition of death receptor/necroptosis signaling and anti-proliferative Gα12/13 pathways, indicating strong capacity for cell survival and proliferation under ART (163). Clonally expanded cells harboring latent provirus can become stochastically reactivated, leading to viral replication of identical sequences derived from multiple anatomical compartments when ART treatment is interrupted (129, 156, 164). Together with data showing that antigen-presenting cells directly enhance latent infection in T cells (165 – 167), these observations argue that tonic signaling, antigenic stimulation, and co-stimulatory signals may regulate HIV reservoir size over time within the CD4^+^ T cell compartment.

Regulation of TCR and co-stimulatory signaling strength, by altering antigen affinity and dose, may dictate whether a particular APC:T cell contact event results in HIV transcription, cell proliferation, or both. Indeed, studies using chimeric antigen receptors (CARs) with different ligand binding affinities showed that receptors with low and intermediate binding affinities did not support proviral transcription, whereas high-affinity CARs facilitated HIV transcription. Notably, strong TCR signals at the time of HIV infection produced inducible latently infected T cells, while weak TCR signals produced latent T cells with irreversible provirus (168). Expression of immune checkpoint molecules is also known to modulate TCR signaling and may favor HIV latency. PD-1, TIGIT, and LAG-3 are preferentially expressed by presistently infected CD4^+^ T cells, where PD-1 can directly silence HIV transcription and reduce their activation potential (112, 151, 169, 170). Elegant single-cell phenotypic analysis of latent T cells also confirmed elevated expression of immune checkpoint molecules PD-1 and TIGIT as well as surface markers associated with cell survival, indicating potential escape from cell-mediated immunity and ability for long-term persistence (171). PD-1 inhibition resulted in HIV latency reversal *in vivo* in both non-human primates and in clinical studies involving PLWH and cancer on ART (172, 173). Recent single-cell RNAseq analysis also describes HIV provirus residing in cytotoxic effector memory Th1-polarized cells with heightened potential to resist cell death (174, 175). Longitudinal immune-mediated host responses can positively select for provirus with low transcriptional activity during prolonged ART, such as those integrated within centromeres (97), indicating ongoing host immune activity against cells that re-initiate viral gene transcription. However, some infected T cells seem to overcome viral cytopathic effects or host immune responses and continue to undergo cell proliferation, directly contributing to the clonal expansion of viral reservoir cells over time (50, 176). These observations argue that infected T cells do not remain transcriptionally silent indefinitely under ART, but rather can cycle between periods of high and low viral production that are subject to host immune pressure. Thus, we postulate that the magnitude of viral transcription is dictated by the strength of antigenic stimulation, cytokine signaling, and inhibitory signals accumulated during T cell transit through various tissues. Findings that viral blips are more common during winter when seasonal viral infections are more frequent (177) and after flu immunization (178) underscores the possibility that repeated microbial antigenic stimulations may favor virus re-activation and/or proliferation of microbe-specific, HIV+ T cells. Additionally, non-circulating CD4 T_RM_ cells at lymphoid and mucosal tissues in humans are clonally distinct from circulating T_EM_ cells in blood, indicating that their antigenic experiences are unique to these environments (99). It is intriguing to speculate that continual pathogen exposure at these sites may drive the clonal expansion of HIV-infected T_RM_ in a manner that is independent of circulating T cell subsets. Hijacking of lymphoid tissue niches that support the long-term replenishment of the HIV reservoir represents a considerable challenge to eradication efforts.

### Concluding remarks and future perspectives

Studies have demonstrated that while many tissues harbor HIV/SIV DNA under ART, secondary lymphoid organs including the lymph node and GALT represent major HIV reservoir tissue sites. These observations suggest that long-lived central memory CD4^+^ T cells containing replication-competent provirus may continue to recirculate between organs, accessing survival signals within specific tissue niches under long-term ART. Notably, continual access to FRC and dendritic cells along collagen networks in the lymph node paracortex, may provide migratory T cells with survival signals during their physiological transit through lymphoid organs. However, how various signals are integrated to maintain the cellular HIV reservoir under ART suppression remains unclear. In addition**,** some thought provoking questions about HIV reservoirs include: (i) do latently infected CD4^+^ T cells retain their capacity to enter and exit lymph nodes as well as their ability to migrate within the lymph node T cell zone *in vivo*, (ii) how is the migratory pattern of latently infected T cell subsets between lymphoid and non-lymphoid tissues tied to the replenishment of tissue viral reservoir, and (iii) whether latently infected T cells are localized within specialized niches within lymphoid organs, and what are the molecular cues that drive their expansion *in vivo*. Some of the possible approaches that may help to address these questions, may include using multi-parameter immunohistochemistry and spatial transcriptomics analysis to define how tissue heterogeneity and gene expression, impacts the genetic profiles of T cells harboring provirus, and also, using two-photon intravital microscopy techniques, to visualize the distribution and migratory behaviors of latent T cells within lymphoid tissues. This may help to identify possible tissue niches that support HIV reservoir longevity. In conclusion, we believe that a better understanding of tissue-derived signals that directly support latent CD4^+^ T cell survival and proliferation may reveal opportunities to therapeutically disrupt such environments to reduce HIV reservoir size in PLWH.
